# Psychophysiological wellbeing in a class of dental students attending dental school: anxiety, burnout, post work executive performance and a 24 hours physiological investigation during a working day

**DOI:** 10.3389/fpsyg.2024.1344970

**Published:** 2024-05-22

**Authors:** Luca Queirolo, Andrea Roccon, Silvia Piovan, Francesco Saverio Ludovichetti, Christian Bacci, Gastone Zanette

**Affiliations:** ^1^Section of Clinical Dentistry, Department of Neurosciences, University of Padua, Padua, Italy; ^2^Department of Philosophy, Sociology, Education and Applied Psychology, University of Padua, Padua, Italy

**Keywords:** anxiety, dentistry, executive functions, psychophysiology, stress, burnout

## Abstract

**Aim:**

To the best of our knowledge, dental school students have never been evaluated for stress, anxiety, burnout, physiological indexes during a 24-h working day, and executive function performance post-work and post-work after returning from vacation; therefore, this research has been conducted.

**Methods:**

Data were acquired at the Dental School of the University of Padua on 16 students in their 4th year, far from the exam session. While performing clinical activity on the dental chair and during a working day, electrodermal activity (EDA), heart rate variability (HRV), and heart rate (HR) were recorded. Participants’ stress was measured with the Perceived Stress Scale (PSS-10 scale) and anxiety with the General Anxiety Disorder Questionnaire (GAD-7) and State–Trait Anxiety Inventory (STAI-Y-2), while burnout with the Maslach Burnout Inventory (MBI-HSS). Executive functions were evaluated using the Tower of London test (TOL-R).

**Results:**

Three students (2F/1M) had a GAD-7 score ≥ 10. Five students (4F/1M) showed trait anxiety. Moderate levels of perceived stress were reported in 85% of participants. MBI-HSS showed that 7 participants scored high on emotional exhaustion and 7 on depersonalization. TOL-R performance (M = 15.85, SD = 4.01) was below the normative value *p* < 0.00001. A second test, after the holidays, showed normal values. EDA was higher during children’s treatment (*p* < 0.05), ANOVA showed high HR during working time (*p* < 0.001), and HRV was higher in males (*p* < 0.001).

**Conclusion:**

Based on the sample size evaluated, it is reported that being a dental student has a moderate impact on stress, anxiety, and burnout while a strong impact on executive functions buffered by rest.

## Graphical abstract

Created with BioRender.

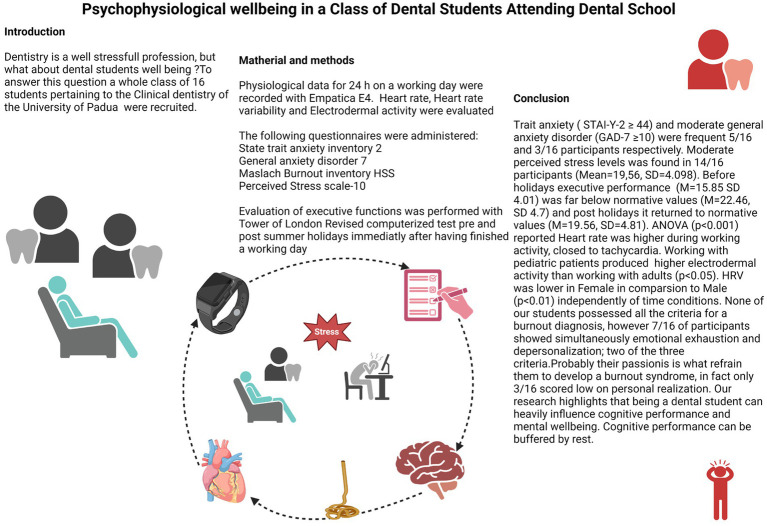

## Introduction

Dentistry is a well-established stressful profession, and the sources of stress and anxiety start occurring while attending dental school. Furthermore, anxiety affects clinical decision-making (Pabst et al., 2013; Chipchase et al., 2017).

Both academic and clinical factors represent causes of stress and anxiety during dental school, and the COVID-19 pandemic led to a negative impact on dental students (Alzahem et al., 2011; Agius et al., 2021). Moreover, Guse et al. found that when comparing dental and medical students, the formers were associated with higher levels of stress, probably due to the higher clinical and practical demands of their academic course (Guse et al., 2021).

Many studies tried to assess stress and anxiety among dental students with validated questionnaires such as the Perceived Stress Scale (PSS), Maslach Burnout Inventory (MBI), and General Anxiety Disorder questionnaire (GAD), and others based their evaluations on customized interview questions with the limit of never supplying objective measures (Alzahem et al., 2011; Smolana et al., 2022). Therefore, the heterogeneity of questionnaires and interview questions used in the different studies does not allow for the reaching of solid conclusions (Elani et al., 2014). Despite these limitations, Deeb et al. found that 40% of dental students in their sample encountered the criteria for burnout subscales (Deeb et al., 2018). The beginning of clinical training and contact with patients are the main stressor agents for dental students (de Souza et al., 2023). Female students report more anxiety than male students, and also the prevalence of trait anxiety seems to be higher in female students (Blumer et al., 2019; Capdevila-Gaudens et al., 2021; Braz-José et al., 2022). Many authors agree on the need for interventions to improve stress and anxiety management, such as counseling programs, as it is a relevant issue also after graduation, being dentistry a highly stressful profession (Gorter et al., 2001; Newton et al., 2006; Basudan et al., 2017).

In addition to questionnaires, a psychophysiological measurement of stress and anxiety can be performed by monitoring psychophysiological variables such as electrodermal activity (EDA), heart rate (HR), and heart rate variability (HRV) (Queirolo et al., 2023). EDA is an index of pure sympathetic activity, which shows the activation of the “fight or flight response,” while HRV is a pure parasympathetic index that is directly proportional to a better ability to manage stress and healthy conditions (Tiwari et al., 2021; Rahma et al., 2022). These indexes are of paramount importance and extensively validated in the literature in relation to stress and affective responses with HRV a little ahead of EDA. Several meta-analyses and systematic reviews support that HRV is an index of wellbeing and is lowered in anxiety or stress-related disorders (Thayer et al., 2012; Cheng et al., 2022; Peabody et al., 2023). EDA, although extensively validated, is still on the rise for ecological applications outside of lab applications (Posada-Quintero and Chon, 2020; Zhu et al., 2023). However, it must be underlined that models for stress detections based on EDA usually reached 82.6% accuracy (Klimek et al., 2023). With limitations of consensus on the threshold adopted and filtering used, there are also some interesting studies that support EDA as the moderator between depression and suicidal ideation in patients with anxiety and depressive symptoms (Sarchiapone et al., 2018; Pruneti et al., 2023). These parameters can be measured with wearable monitoring devices and can provide data for psychological-related issues (Hickey et al., 2021) and the evaluation and monitoring of work-related performance (Bhoja et al., 2020; Claverie et al., 2020). Particularly, psychophysiological variables are gaining interest in the assessment of stress and burnout in the field of occupational medicine (De Looff et al., 2018). Recent findings in Dentistry suggest that they can also supply information to assess dental anxiety in patients (Drury and Simonetti, 2019; Košir et al., 2021; Torres-Gomez et al., 2021).

The aim of this study is to examine psychophysiological parameters during working hours, psychological data, and executive functions in dental students, comparing their physiological parameters during the day (working hours activity, daytime, i.e., non-working hours activity, and sleep), and investigate if there is any difference between those working with adults (C.rossa group) and those working with pediatric patients (Treviso group). Furthermore, as a secondary outcome, this study aims to explore gender differences according to physiological or psychological data.

## Materials and methods

Psychological data and physiological measures were recorded from 16 healthy dental students attending the Dental School of the University of Padua (Padua, Italy) between 31 October 2022 and 22 December 2022 for the first part, far from exam sessions and the retest of TOL-R after summer holidays far from exam sessions. Exclusion criteria were cardiovascular diseases or psychiatric disorders. A diary with relevant information was also compiled by participants to assess if there would have been any excessive stress during the day or working activity. The inclusion criteria were being a dental student at the Dental Clinic of the University of Padua, attending the pedodontics unit, or a general dentistry office with adult patients. The age of participants (11 females and 5 males) ranged from 23 to 32 years. This study is composed of a mixed design: a within-participants study and a between-subjects study. The physiological activity of each participant was evaluated according to three periods: working time, daytime, and sleep during 24 h. Furthermore, students were also divided into two groups: 8 worked in the pedodontics office with pediatric patients (Treviso group; younger than 14 years) and the other 8 spent the working day in a general dental office (C.rossa group; with adult patients). Written informed consent was obtained from all the participants. Data were acquired between September 2022 and January 2023. The study was approved by the Ethical Committee of Azienda Ospedale—Università di Padova, n. 278n/AO/23.

### Evaluation of stress

The stress levels of participants were measured through the Perceived Stress Scale (PSS-10). Scores ranging from 0 to 13 are considered low stress. Scores ranging from 14 to 26 are considered moderate. Higher than 26 would be considered high perceived stress. This scale reflects the perceived stress levels in the last month.

### Evaluation of anxiety and burnout

Anxiety levels of participants were measured with the State–Trait Anxiety Inventory Y2 (STAI-Y2) (Spielberger, 1989) and the Generalized Anxiety Disorder questionnaire (GAD-7) (Spitzer et al., 2006). Maslach Burnout Inventory HSS questionnaire was also administered to participants (Maslach and Jackson, 1981). The subjects can be defined as anxious with STAI-Y-2 scores equal to or greater than 44, while regarding the GAD, different thresholds define normal, moderate, or severe generalized anxiety. The GAD value that the literature describes as the cutoff for a diagnosis of generalized anxiety disorder is a value equal to or greater than 10. Burnout is a multidimensional phenomenon. Suggestion of possible burnout syndrome is suspected when someone shows high levels of emotional exhaustion and depersonalization accompanied by low levels of personal realization (Maslach and Jackson, 1981; Maslach, 1993). MBI-HSS Italian’s normative data suggest high emotional exhaustion when the scores are equal to or above 23, high depersonalization when scores are up or above 6, and low professional realization when scores are lower or equal to 31 (Sirigatti and Stefanile, 1993).

### Evaluation of cognitive performance

The Tower of London test (TOL) at the end of a regular day of work and after the summer holidays is the only evaluation that was repeated to explore if resting activities could buffer their very low executive performance in the first instance. This cognitive test evaluates executive functions, a set of modules very sensitive to excessive stress that regulate the cognitive system’s planning, control, inhibition, and coordination processes (Schnirman et al., 1998; Boccia et al., 2017).

### Physiological measures

Physiological parameters were obtained using Empatica E4, a wearable device in the form of a bracelet that measures electrothermal activity (EDA), blood pulse volume—from which heart rate (HR) and heart rate variability are derived (HRV)—temperature, and movement (Garbarino et al., 2014). EDA is a property of the skin that highlights the variation of electrical conduction in response to sweat secretions and is a pure sympathetic index (Boucsein, 2012). HRV is the physiological variation of the time interval between heartbeats. It is a parasympathetic index that reflects vagal activity and is measured by the root mean square of successive differences between the beat intervals (Shaffer et al., 2014; Ernst, 2017; Kim et al., 2018). This monitoring device was worn by students on their non-dominant hands for 24 h during a working day, and they were asked to report the start and the end of their activity during the day. The 24 h were divided into three monitoring periods called conditions: “work,” “sleep,” and “daytime.” We assume that daytime temporization reflects a similar physiological activity to a non-working day. HR was expressed in beats per minute (bpm) and derived through Empatica algorithms from the blood volume pulse. From inter beats intervals (IBI) and photoplethysmography (PPG) signal, HRV was extracted as the root mean square of successive differences between normal heartbeats (RMSSD) by first calculating from IBI each successive time difference between heartbeats in *ms*, and then over a short-term period of 30 s. Then, each squared and averaged value was obtained before the square root of the total was derived. The sensor used to detect blood volume pulse is a PPG sensor, which is known to be subject to missing data due to movement/pressure artifacts (Chen et al., 2015). Artifacts were removed, discarding zero values and other single data point outliers. The analysis of EDA resulted in the extraction of the skin conductance level. The electrodes used were silver coated with copper underlay on brass electrodes. The threshold for the amplitude of the significant signal was set to a minimum rise of 0.005 μSiemens. EDA values were then normalized using the min-max method. Physiological data were down-sampled to 1 Hz and labeled as belonging to condition “work,” “sleep,” or “daytime.” Data of each subject were then aggregated into periods and groups expressing their mean values.

### Statistical methods

As a first step, the characteristics of the subjects with anxiety were compared with those without anxiety using dependent t-tests and a chi-squared test. We then next evaluated whether working with children or adults presents some differences. Then, we evaluate if physiological parameters (independently of working setting) differed between subjects during the three periods of the day (“condition” factor, 3 levels, within-subjects), according to anxiety symptoms reported using GAD-7 and STAI-Y2 (factor “anxiety,” 2 levels, between subjects) or gender differences (factor “gender,” 2 levels, female or male). The analyses were performed with R version 3.5.1, which was also used to test the hypothesis of normally distributed residuals on all levels of the model. Separate models were considered for each parameter type (EDA, HR, and HRV).

## Results

Welch’s *t*-test and the *χ*-squared test related to EDA, HR, and HRV according to STAY or GAD-7 values did not show any significant outcome. Pearson correlation test analyzing anxiety or stress and executive performance did not show any significant outcome.

### Stress

Fourteen participants showed moderate stress perceived levels, 1 participant showed high perceived stress levels, and only 1 subject scored low on the stress perceived scale (Mean = 19,56, SD = 4.098). The diaries showed five stressful complaints during working activity compared to 7 in non-working activity.

### Anxiety

Approximately 31.25% of participants (4 F, 1 M) showed levels adequate for trait anxiety diagnosis measured with the STAI-Y2 questionnaire (mean = 40.5625, SD = 7.8057). The two groups (adult/pediatric patients) did not show differences in the distribution of trait anxiety. The chi-square statistic (Anxiety-Gender) was not significant. GAD questionnaires revealed that three subjects (2 F, 1 M) showed levels of anxiety above the cutoff for the diagnosis of a moderate generalized anxiety disorder (GAD-7 ≥ 10).

### Burnout

Five participants showed both high levels of emotional exhaustion and depersonalization. Seven participants showed high levels of emotional exhaustion, 7 showed high levels of depersonalization, and 3 showed low levels of personal realization. None showed high emotional exhaustion, depersonalization, or low levels of personal realization.

### Electrodermal activity

**Figure 1:**
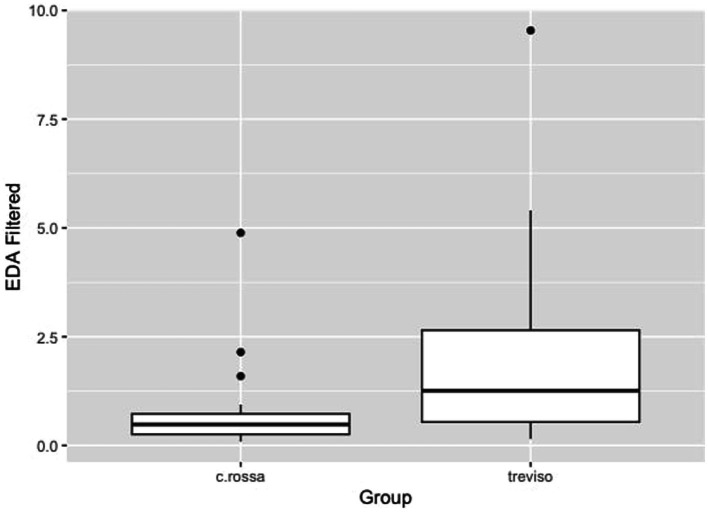
Electrodermal activity, EDA, comparison between Croce Rossa group (dental student dealing with adults) and Treviso (dental student dealing with children) *p* < 0.05.

Welch’s two-sample t-test showed a significant difference (*t* = −2.6598, df = 32.593, *p*-value = 0.01203, 95% confidence interval − 2.3586374 to −0.3136082) among EDA values of students in the dental office working with adults (mean = 0.7474038) and pediatric patients (mean = 2.0835266) (Figure 1).

### Heart rate

**Figure 2:**
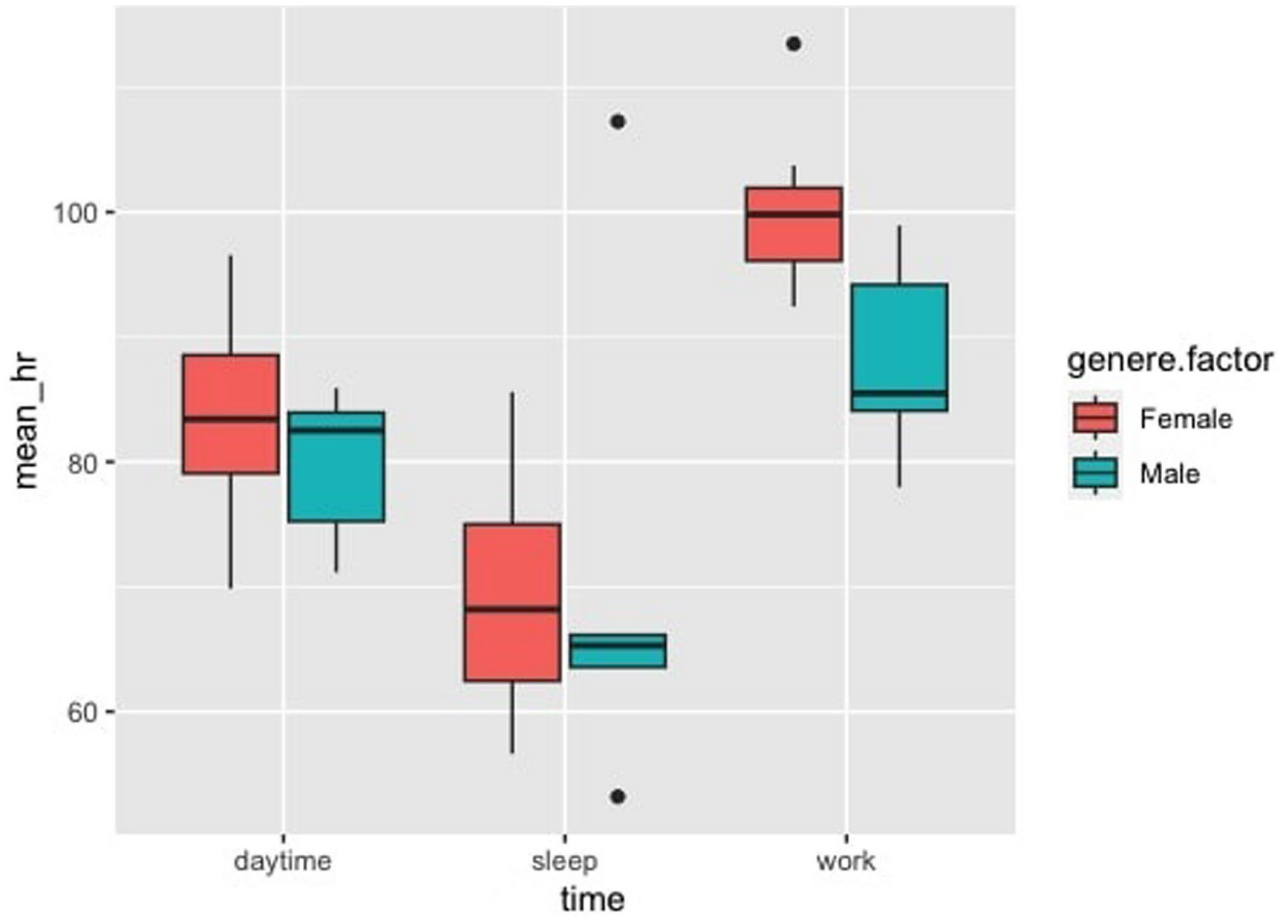
Heart rate, HR, according to anxiety levels and conditions: no effect have been found for anxiety levels on physiological data, but there is a difference between HR in relationship to work vs. sleep and work vs. daytime, *p* < 0.001.

Welch’s two-sample t-test did not show a significant difference between the two groups (adults/pediatric patients). The ANOVA showed an effect of daytime versus night-time status independently of group pertinence, sex, or anxiety: heart rate during working hours was higher than during sleep and non-working daytime status (*p* < 0.001) (Figure 2).

### Heart rate variability

**Figure 3:**
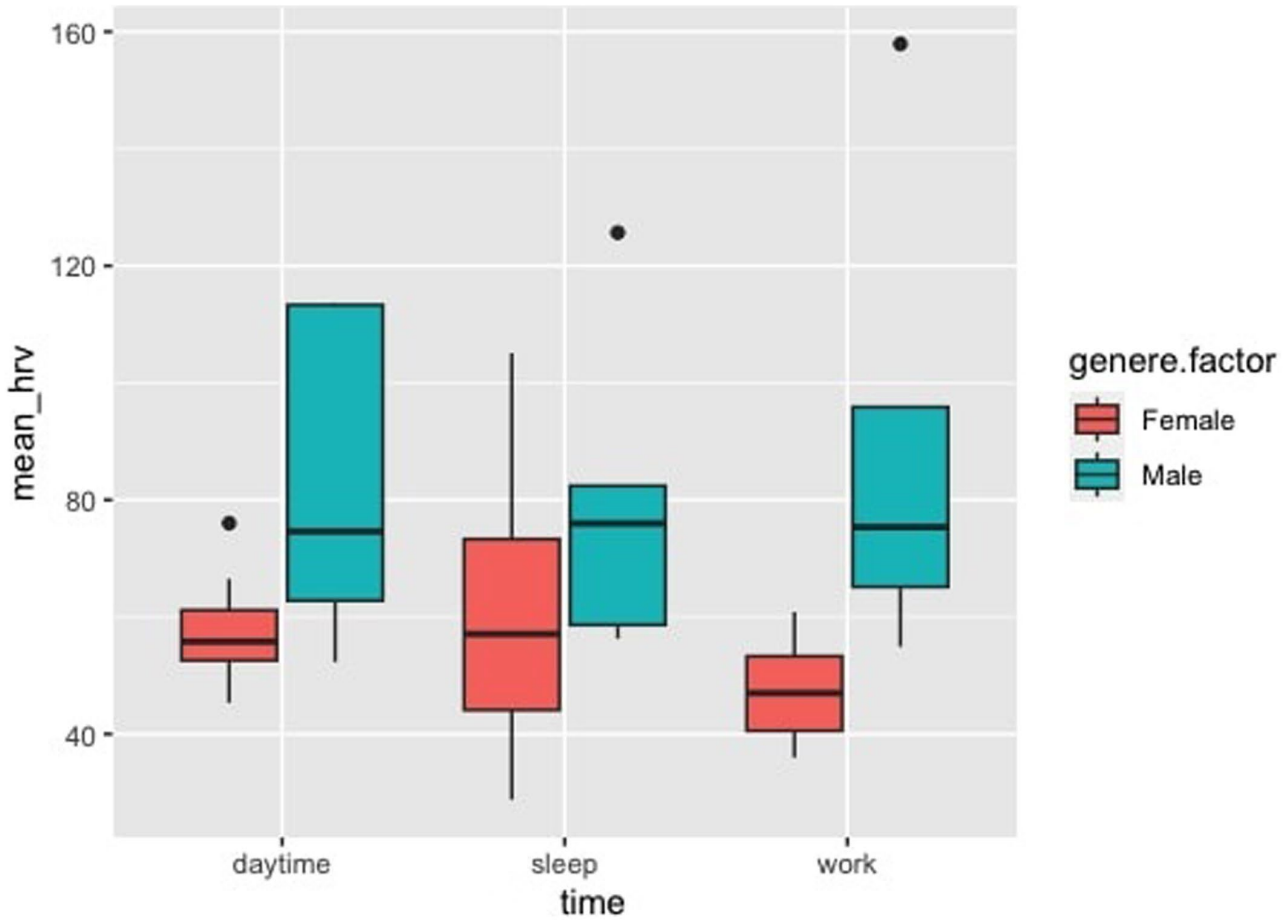
Heart rate variability, HRV. Analysis of variance in relationship to being female or male in three different times: daytime = non-working activities, sleep, and working activities = dental students working in the Dental Clinic. It is possible to appreciate that male independently of conditions express higher parasympathetic activity, *p* < 0.001.

Welch’s two-sample t-test did not show a significant difference between the mean HRV values of the two groups (adults/pediatric patients). On the other hand, the ANOVA showed a higher parasympathetic tone in men (*p* < 0.001) than in women regardless of working conditions (day, work, and sleep) (Figure 3).

### Cognitive performance

**Figure 4:**
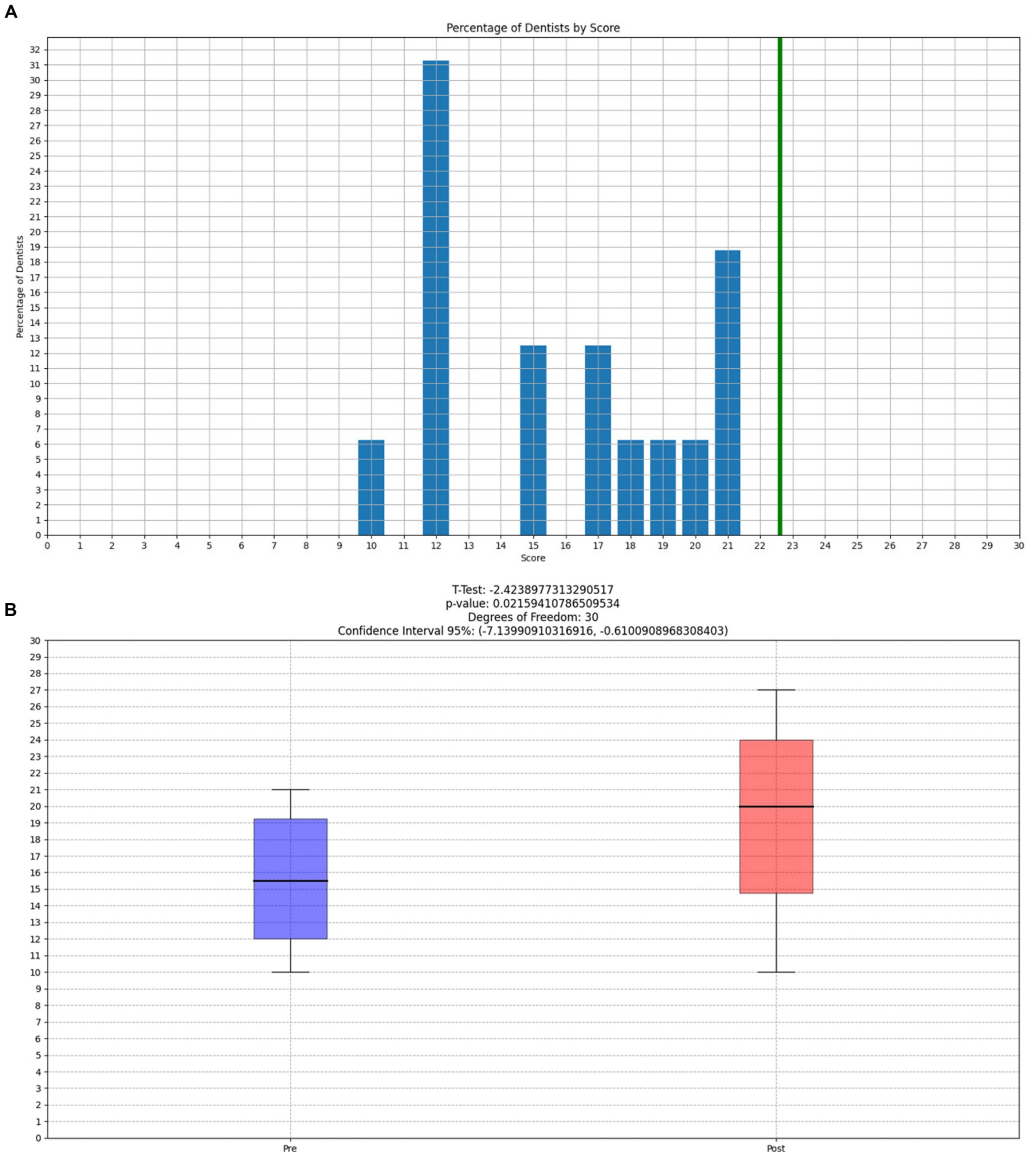
**(A)** Dentists’ Tower of London Revised (TOL-R) score, showing the densities of post-work scores. The Green line represents the mean of normal Normative value for TOL-R (22.6). Participants showed a mean of 15.87 and a comparable SD in comparison to normative values 4.87 vs. 4.01. From this image, it is possible to appreciate that no participant reaches the mean of normative value. **(B)** T-test between pre-holidays and post-holidays Tower Of London-Revised (TOL-R) cognitive performance.

Cognitive performance at the Tower of London test at the end of a working day was deficient; 13 out of 16 subjects performed below-average scores (Mean = 15.87, SD = 4.01) and 62% of our sample showed a performance at least below 1.5 SD and 37% more than 2 SD below average (Figure 4). TOL-R validated values are 22.6 with SD = 4.7, and it has shown to be psychometrically superior about reliability in comparison to TOL (Schnirman et al., 1998). Given the result, we also investigated participant commitment to doing the test, and only one dental student declared to have provided a low effort doing the test. Therefore, we administered the TOL-R also after the summer holidays to understand if this drop in cognitive performance was due to excessive workload as suggested by the questionnaires. Data showed that cognitive performance, after holidays, returned to normal value (Mean = 19.56, SD = 4.81). We also checked with the F-test for equality of variances (after checking the Kolmogorov–Smirnov normal distribution of our TOL-R scores). Our sample population’s variance ratio is considered equal to the TOL-R population’s variance ratio, the *p*-value was 0.5242 (*p* (*F* ≤ 1.3737) = 0.7379), and *F* = 1.3737 in the H0 regions of acceptance [0.4301, 2.6517]. Therefore, their low performance cannot be explained by a difference in variance between the two samples, but they pertain to two different groups with the same variance. Given the assumption, we calculate a one-tail T-test to evaluate the magnitude of the difference between TOL-R validation values and the data provided by our sample. The values from TOL-R validation are greater than those of our sample *p* < 0.00001 T = 5.1059 region of acceptance H0 [−inf., 1.682], the effect size d is large 1.58, indicating that the magnitude of the difference between the averages is large.

## Discussion

Several studies tried to measure stress in dentists with both objective and subjective parameters (DiMatteo et al., 1993; Moore and Brødsgaard, 2001; Myers and Myers, 2004; Newton et al., 2006; Bodner, 2008; Tammayan et al., 2021). Very few have tried to investigate the physiological and psychological activity in an entire working day (Queirolo et al., 2023). However, no study, to the best of our knowledge, has ever addressed a class of young students in a holistic evaluation through the psychological, physiological, and executive functions conundrum.

Starting from the analysis of psychological data, the fact that working with patients is an anxiety-producing event is confirmed (Enkling et al., 2006). Significant is the fact that as many as 31.25% of dental students have an STAI-Y2 score that demonstrates trait anxiety, in agreement with the largest meta-analysis carried out in 2019 (Quek et al., 2019), which showed anxiety disorders in 33.8% of medical students (95% confidence interval, 29.2–38.7%). The prevalence of generalized anxiety disorder in our sample was 18.75%, while among the medical professionals, it ranges from 2.8 to 8.5% and from 1.6 to 5% in the general population (Leon et al., 1995; Olfson et al., 1997; Roy-Byrne and Wagner, 2004; Kessler et al., 2005).

The two groups showed different sympathetic activities, as shown in Figure 1. Working in contact with children (Treviso group) caused greater sympathetic activity, probably because relating to a child is more complex than with a collaborating adult (C.rossa group). In the within-participants analysis, heart rate was shown to be different in the working condition compared to both the non-working daytime condition and the sleeping condition, with average levels close to tachycardia in the working condition (Figure 2). This demonstrates the considerable cardiovascular effort these young students are subjected to. It should be remembered that a higher heart rate is associated with a higher chance of death from both cardiovascular and non-cardiovascular causes (Woodward et al., 2014; Alhalabi et al., 2017).

The Tower of London test showed below-average scores, with a small number of subjects (2) meeting the normative values at the end of a working day (Figure 4). This has huge implications demonstrating how much stress, subcomponents of burnout, and anxiety can alter planning ability and mental organization. Executive skills are the hallmark of the functioning of the prefrontal cortex. The dependence of cognitive fatigue due to work is also confirmed by normal values post-holidays. A higher level of stress corresponds to worse performances in identifying and treating caries and/or other oral pathologies. Until 2018, no one cared about this relationship when Plessas et al. highlighted a “gap” in the literature (Plessas et al., 2018). A subsequent study filled the pre-existing “gap” demonstrating that stress measured with VAS in relation to time pressure is an important marker of poor clinical performance (Plessas et al., 2019). Using anxiety or perceived stress, we did not find any significant correlation with executive functions; nonetheless, their executive performance was below normative values, and they have already demonstrated moderate stress and a high prevalence of general anxiety disorder and anxiety. The secondary outcome, to investigate the presence of gender differences, has reported that female students had a lower parasympathetic tone regardless of the condition analyzed (daytime, work, and sleep), as shown in Figure 3. This finding contrasts with the current literature, which shows a higher parasympathetic tone in female than in male (Koenig and Thayer, 2016).

The study we have conducted concretely demonstrates that a lower cognitive performance is associated with stress biomarkers (lower HRV in Females, higher EDA in dental students working with pediatric patients, and higher HR in all groups during working time).

The limited sample suggests being cautious to generalize the data to the general population; furthermore, it is difficult to understand what has contributed to the actual situation. Longitudinal studies, therefore, are needed. Another weakness resides in the fact that no correlation between anxiety or stress and executive performance was found; for anxiety, this probably resides in sample limitations, while for stress, it is because all subjects scored high in their perceived stress levels and were quite homogeneous in this sense; furthermore, the distribution of perceived stressful events was quite the same across the working day and non-working day activities and above all, no complaint suggesting a non-routinary day of work has been reported by participants.

The data presented in this study highlight the complex relationship between stress, anxiety, burnout, and executive functions. Our data showed that general anxiety disorder and trait anxiety appear to be very common in dentistry and can be found in dental school attendance periods. Quite all participants referred to moderate perceived stress levels, but more than half showed a performance in executive function far lower than expected, highlighting the difference between the perception of stress and performance in people perceiving being moderately stressed. This study also highlights that subcomponents of burnout can be detected already from attending dental school. In comparison with the data presented by Deeb, our research on a whole class of dental students highlights 43.75% of emotional exhaustion and the same percentage for depersonalization, and we also evaluate personal realization (18.75% of our sample showed low personal realization) that relates to mastery and social connections. We did this investigation because burnout can be buffered by personal realization; in fact, being in an environment that promotes development and a caring and positive attitude can significantly impact stress-related issues (Karasek, 1979; Karasek and Theorell, 1990). Although based on a small sample size, our study underlines that burnout is a multidimensional construct and that the data presented by Deeb suffer from inflation because of methodological issues: proxy questions, partial evaluation where personal realization was not taken into account, and data of participants with all the three subcomponents of burnout were not provided (it is necessary to meet all the three subcomponents criteria for a burnout diagnosis). This data supports the necessity of psychological counseling and the urge to implement stress management, which has been shown to be useful in the general population (O’Toole et al., 2018; Vos and Vitali, 2018; Nakao et al., 2021), in dentists (Chapman et al., 2017), and probably also in young dental students.

## Conclusion

From the data, although on a limited sample, appears that a dental student loving his/her future job can buffer the effect of emotional exhaustion and depersonalization on self-perceived stress to manifest the burnout syndrome. However, if dental students are stressed, personal realization cannot alter the deficit in goal-oriented behavior, i.e., it is confirmed that performance is affected by fatigue or stress and loving his/her job is not enough to buffer and therefore refrain from poor executive performance. A deficit in those skills that allow an individual to anticipate, plan, set goals, implement goal-directed projects, monitor, and self-regulate their behavior to adapt it to new conditions is of paramount importance because being a dentist requires these functions as a prerequisite. It is very important to underline that executive performance went back to normative values after the holidays, highlighting the relevance of rest. The interplay between cognitive workload (deficient performance at the Tower of London revised), anxiety, burnout, and stress is supported also on a physiological basis as the HR during working day was higher than daytime HR. Dentists working with children and, more specifically, females appear to be more at risk. This is the consequence of a higher sympathetic activity, a higher heart rate, and a lower parasympathetic tone. From a general overview, it is possible to sustain that being a dentist is stressful both from a psychological as well as a physiological perspective. All these data show the urge for psychological counseling, training in stress management, the importance of a global approach that can allow for improving performance through improving subjective wellbeing, and prove the relevance of rest.

## Data availability statement

The raw data supporting the conclusions of this article will be made available by the authors, without undue reservation.

## Ethics statement

The studies involving humans were approved by Ethical Committee of Azienda Ospedale – Università di Padova, n. 278n/AO/23. The studies were conducted in accordance with the local legislation and institutional requirements. The participants provided their written informed consent to participate in this study.

## Author contributions

LQ: Conceptualization, Data curation, Formal analysis, Investigation, Methodology, Writing – original draft. AR: Writing – original draft. SP: Writing – review & editing. FL: Writing – review & editing. CB: Writing – review & editing. GZ: Project administration, Supervision, Writing – review & editing.
